# Integrating Nutrition Actions in Service Delivery: The Practices of Frontline Workers in Uganda

**DOI:** 10.34172/ijhpm.2022.5898

**Published:** 2022-04-24

**Authors:** Brenda Shenute Namugumya, Jeroen J.L. Candel, Elise F. Talsma, Catrien J.A.M. Termeer, Jody Harris

**Affiliations:** ^1^Public Administration and Policy Group, Wageningen University & Research, Wageningen, The Netherlands.; ^2^Division of Human Nutrition and Health, Wageningen University & Research, Wageningen, The Netherlands.; ^3^Institute of Development Studies, University of Sussex, Brighton, UK.

**Keywords:** Nutrition Practices, Street-Level Bureaucracy, Nutrition Policy, Policy Integration, Uganda

## Abstract

**Background:** Integrating nutrition actions into service delivery in different policy sectors is an increasing concern. Nutrition literature recognizes the discrepancies existing between policies as adopted and actual service delivery. This study applies a street-level bureaucracy (SLB) perspective to understand frontline workers’ practices that enact or impede nutrition integration in services and the conditions galvanizing them.

**Methods:** This qualitative exploratory study assesses the contextual conditions and practices of 45 frontline workers employed by the agriculture, health and community development departments in two Ugandan districts.

**Results:** Frontline workers incur different demands and resources arising at societal, organizational, and individual level. Hence, they adopt nine co-existing practices that ultimately shape nutrition service delivery. Nutrition integration is accomplished through: (1) ritualizing task performance; (2) bundling with established services; (3) scheduling services on a specific day; and (4) piggybacking on services in other domains. Disintegration results from (5) non-involvement and (6) shifting blame to other entities. Other practices display both integrative and disintegrative effects: (7) creaming off citizens; (8) down prioritization by fixating on a few nutrition actions; and (9) following the bureaucratic ‘jobs worth’. Integrative practices are driven mostly by donors.

**Conclusion:** Understanding frontline workers’ practices is crucial for identifying policy solutions to sustain nutrition improvements. Sustaining services beyond timebound projects necessitates institutionalizing demands and resources within government systems. Interventions to facilitate effective nutrition service delivery should strengthen the integrative capacities of actors across different government levels. This includes investing in integrative leadership, facilitating frontline workers across sectors to provide nutrition services, and adjusting the nutrition monitoring systems to capture cross-sector data and support policy learning.

## Background

**Key Messages**

** Implications for policy makers**

- Learning from lived experiences offers important insights of the micro-contextual processes and patterns that can be used to improve how integrated nutrition policies are practiced on the ground.
- Without donors projects putting pressure on and facilitating frontline workers, nutrition integration into services delivery would remain challenging to realize and sustain. Sustaining services beyond timebound projects necessitates institutionalizing nutrition policy demands and resources within government systems.
- Facilitating frontline workers from different sectors in the implementation of nutrition policies is important to foster collective response. Developing integrative leadership capacities, designing multi-sector nutrition indicators and monitoring systems, and facilitating cross-sector policy learning can help in this facilitation of work.

** Implications for the public**

Integrated nutrition strategies (INSs) are policy responses to facilitate collective action by actors across government sectors and levels of operation to improve nutrition in Uganda. We demonstrate that understanding the integrative practices of different frontline workers presents vital opportunities to identify effective policy solutions to facilitate integrated government action to improve and sustain nutrition outcomes. The study reveals various interacting context-specific conditions – individual, organizational, societal; and the resultant practices adopted by frontline workers to structure and modify nutrition services delivery. This important to inform advocacy for explicit long-term investment in nutrition service delivery by all relevant government ministries and across governance levels.

There is global consensus on the need to integrate nutrition-related actions into the service delivery systems of different policy sectors as a measure toward the sustainable reduction of malnutrition and its effects.^1,2^ Following the Scaling Up Nutrition Movement agenda, governments and international actors have invested in developing integrated nutrition strategies (INSs), which aim to create an enabling policy environment that: (*i*) inspires the continued incorporation of nutrition objectives and instruments into the policies of different sectors (eg, health, education, agriculture); (*ii*) facilitates different organizations to prioritize nutrition actions in service delivery, and (*iii*) enables the convergence of goods and services from different sectors in the same households and individuals.^3^

 Despite these ambitions, there exists considerable discrepancies between nutrition policies as adopted and the ultimate delivery of nutrition services,^4^ with potential consequences for the attainment of policy goals. Policy science studies show that integrated policy strategies often fail to be delivered as intended,^5^ and call for more refined understanding of how integrative demands are experienced and molded into action on the ground.^6^ Currently, limited nutrition studies exemplify what *actually* happens during implementation. This specifically concerns the dynamics of integrating nutrition actions into the daily operations of frontline workers.^7-9^ Frontline workers are employees of public and private sector entities who interact directly with citizens and have wide discretion over the allocation or sanctioning of benefits associated with their daily work.^10^ Additionally, there are minimal insights about how frontline workers of the sectors considered as *new* entrants (eg, agriculture, social development, education) to this policy issue deliver services. Though, the frontline workers’ actions are affected by various contextual conditions,^11,13^ few systematic studies compare these conditions across policy sectors and implementation contexts.

 There is increasing pressure on countries to implement the multi-sectoral strategies at scale, involving different government departments (horizontal integration) and government levels (vertical integration), to sustain the positive changes in their nutrition situation. It is therefore important to examine how integrated nutrition action materializes in service delivery. One way to expand our understanding is to reflect on the practices of frontline workers offering services to citizens. Thus, this research investigates: *what practices are adopted by frontline workers to either enact or impede nutrition integration during service delivery, and the contextual conditions that galvanize them*. Recognizing these practices and their activating conditions is important for monitoring the effectiveness of nutrition policies and for focusing efforts that design strategies to sustain nutrition integration into service delivery.

 We adopt a bottom-up implementation perspective by applying the street-level bureaucracy (SLB) approach to explore frontline workers’ everyday practices. The SLB approach argues that frontline workers confront various pressures from multidimensional contexts, including the institutional and socio-political environment, that influence them to make discretionary decisions and establish coping routines which effectually show how policies are translated during implementation.^10^ This implies that variations in the implementation context shapes services delivery in different ways.^13^ We study frontline workers’ practices and the conditions influencing them for insights about nutrition integration/disintegration on the ground. This study identifies and compares the practices of the frontline workers expected to deliver nutrition services in Uganda.

 Uganda loses approximately 5.6% of annual gross domestic product because of undernutrition.^14^ Despite childhood stunting decreasing to 29%, regional variations range between 14% and 41%. Other nutrition indicators show similar regional disparities.^15^ The country has endorsed INSs since 1996 to guide concerted actions of different actors tackling malnutrition. Recent studies indicate some shifts toward increased nutrition policy integration across ministries,^16,17^ making Uganda a good case to explore how integrative demands to improve nutrition are enacted or impeded by frontline workers.

###  Operationalizing Integrated Government Action and Street-Level Bureaucracy 

 Theoretically, successful policy integration processes is expected to result in *integrated government action* during service delivery.^6,18^ The concept is discussed under different labels, including joined-up government,^19^ whole-of-government,^20^ and multisectoral action.^21^ We define integrated government action as the continuous efforts of actors in different policy sectors to holistically improve the responsiveness and effectiveness of services provided to citizens to reduce malnutrition.^22^ This means that the INSs are expected to inspire interactions among diverse actors, across policy sectors and governance levels; and to ensure these policies become continually and consistently translated into specific nutrition objectives and interventions for implementation.

 Most studies on integrated government action have focused on the top-down alignment and coherence of policies, institutions, and administrative operations,^5,23^ which infer government’s capacity to realize policy goals. However, it is extensively argued that the practices observed during implementation are determined by various conditions interacting in a given context, rather than by policy prescriptions alone.^9^ INSs are not automatically adequate to galvanize nutrition integration in service delivery. Hence, the need for a bottom-up approach to identify the actual practices of the frontline workers expected to provide nutrition services.

 We apply the SLB perspective to examine how and why integrative demands are or are not realized on the ground. This perspective suggests that frontline workers encounter varying conditions during service delivery that lead them to establish routine practices that shape how citizens experience policies.^10^ We define practices as the habitual actions and behavior efforts employed by frontline workers during their day-to-day work interactions with citizens. That is, the ways in which frontline workers negotiate work circumstances to allocate − or withdraw − nutrition services.^10^ On the one hand, ideal practices for frontline workers may be identified from the nutrition literature which distinguishes several nutrition-specific and nutrition-sensitive interventions.^24-26^ In brief, nutrition-specific interventions address the immediate determinants of fetal and child nutrition and development; and nutrition-sensitive interventions focus on the underlying determinants of good nutrition such as good governance and sufficient resources. Several nutrition studies highlight the interventions offered and the gaps in service delivery. However, focusing on interventions alone is too limiting to represent the complex and dynamic interactions practices that determine how service delivery works.

 On the other hand, Lipsky^10^ differentiates three main patterns of practices developed by frontline workers to avert confrontations associated with encountered work pressures. These are: first, practices that limit citizens’ demands and maximize the use of available resources, such as queuing citizens, withholding information, and limiting access to personnel. The strategies enable the structuring of how services are distributed.^27^ Second, practices that modify frontline workers’ understanding of their job expectations so as to align with available resources to achieve targets, like preferential selection of citizens and favoring speed over need.^10,28^ These practices potentially control the supply of services. Third, practices that modify citizens’ perceptions of available services including rubber stamping judgements and drawing boundaries. This permits frontline workers to make the gaps between accomplishments and objectives acceptable.^29^ Generally, this categorization implies that nutrition integration into service delivery is shaped in divergent ways.

 Several SLB studies rationalize that frontline workers adopt practices to manage the imbalances usually incurred between the demands to act (action prescriptions) and the resources available to realize them (action resources).^13^ This literature synthesizes various multidimensional contextual conditions, depicting the demands and resources that interact at individual, organizational, and societal level to determine services. Table 1 distinguishes the conditions with potential to influence the responses to integrative demands.

**Table 1:** Conditions Shaping How Frontline Workers Integrate/Disintegrate Nutrition Services

**Levels**	**Action Prescriptions (Demands)**	**Action Resources **
Society (sub-county, district, national socio-political)	Policies (eg, INSs)	Information
	Development actors (ideologies, expectations)	Nutrition budgets
	Citizens’ expectations	Training (knowledge)
	Performance measures	
Organization (department, ministry)	Policies (guidelines)	Training (knowledge)
	Performance measures	Information (guidelines)
	Professional norms and conduct	Nutrition budgets
	Supervision	Management
Individual	Competing job tasks (time)	Education (competences)
	Expertise of frontline worker	Professional experience
	Client numbers	Freedom to decide (discretion)
	Personal beliefs and values	Incentives for working

Abbreviation: INSs, integrated nutrition strategies. Adapted from Hupe and Buffat,^13^ modified by the current authors based on the nutrition literature.

 Regarding individual-related conditions, frontline workers have varied job descriptions, attitudes toward citizens, expertise, and perceptions of appropriate behavior. The conditions are further influenced by participation in professional and social networks.^30^ Increasing the frontline workers’ knowledge of integrative demands, and the associated professional gains, may facilitate service delivery.^31^

 Organization-related conditions are determined by the administrative and management structures to which frontline workers are obligated to account. An organization’s capacity to influence its workers to integrate an issue depends on its mandate, collective beliefs, structure, and resources required.^32^ For example, organizational measures may contribute to legitimizing or excluding certain practices of frontline workers.^27,28,33^ That is, whereas guidelines may increase similarities in adopted practices, and managerial supervision strengthens alignment using rules (integrative demands), resource constraints are associated with reduced task prioritization (disintegration).^13^

 Societal-related conditions comprise of prerequisites from administrative superiors and international actors, existing policy ideologies, and client caseload. The conditions inevitably affect both individual- and organization-related factors.^28,34^ Though nutrition policies in African countries are largely influenced by international organisations,^35-37^ the actors have different perspectives for realizing development which may be reflected in the practices adopted on ground. High financial investment is expected to motivate nutrition integrative practices.

 In this study, we examine the individual-, organization-, and societal-related conditions that explain the nutrition integrative/disintegrative practices of frontline workers. These conditions are expected to differ within and across policy jurisdictions and geographic boundaries, thereby enabling comparison of the practices of different frontline workers. Identifying the actual conditions propelling the emergence of desired and undesired practices is crucial for designing solutions to facilitate nutrition integration in service delivery systems.

## Methods

###  Research Design 

 Few empirical studies investigate the daily lived experiences of frontline workers who implement INSs in low-income countries.^4^ The qualitative exploratory research applies an interpretive approach which permits the identification of activities and the interactional patterns to construct how observed outcomes develop. Using qualitative inquiry methods, we conduct a comparative analysis of the services offered in two districts in Uganda − Moroto and Namutumba to reveal comprehensive details about the contextual conditions and the routines practices that explain the dynamics shaping the disparities in nutrition integration.

###  Study Context 

 We selected the districts based on the prevalence of malnutrition measured by stunting and investment in nutrition programs reflected in the presence of international actors namely international non-governmental organization (NGOs) and donor projects. Childhood stunting is the main nutrition indicator prioritised in the Uganda Vision 2040 and the Uganda Nutrition Action Plan 2011-2016. Since the 1960s, Moroto has continued to have several international actors implementing projects to address the persistent high prevalence of undernutrition (ie, stunting, wasting and underweight). Childhood stunting rate for Moroto is 33%.^38^ In addition to the government budget, Moroto has several active nutrition projects supported by donors, such as United Nations agencies and the United States Agency for International Development is (USAID).^39^ In Namutumba, the childhood stunting rate is 28%.^40^ Most district operations rely solely on government funding. The area has only three small scale nutrition-focused projects supported by World Bank and USAID. Our focus is limited to the frontline workers in the government departments because they are mandated to improvement government policies. of health, agriculture, and community development.

###  Data Collection Methods and Analysis

 The primary respondents comprise the frontline workers in the departments of health, agriculture, and community development representing all the sub-counties in each district (Table 2). Prioritization of the departments was informed by the Uganda Nutrition Action Plan which highlights the departments considered are necessary to implement the policy at subnational level.^41^ Further, recent research shows a general shift toward increased nutrition integration in the policies developed by the ministries of health, agriculture and community development.^16^ To ensure that the typical practices of frontline workers are captured, we purposively selected the health workers operating in health centres III and II in Uganda’s health system structure. Interviewees included all health workers assigned the nutrition-focal person duties. In addition we interviewed all agriculture extension workers (AEWs) and community development officers (CDOs) employed at district and subcounty levels. To gain more understanding of o the contextual conditions shaping nutrition services, we interviewed the sub-counties and districts management officials, and the nutrition staff of NGOs implementing nutrition-related projects in the two districts. The sampling approach and selection of respondents is elaborated in Supplementary file 1.

**Table 2:** Summary of Respondents

**Level of Operation**	**Categories of Respondents**	**Respondents Per District**		**Area of Focus**
		**Moroto**	**Namutumba**	
Frontline workers (service delivery)	Health workers	12	14	Practices and contextual factors
	AEWs	5	4	
	CDOs	5	5	
Administration (government)	Sub-county	6	5	Contextual factors (organization and societal)
	District	4	4	
Non-government actors (donors, INGO, CBO)		4	3	
Total respondents		36	35	

Abbreviations: CBO, community based organization; INGO, international non-governmental organization; AEWs, Agriculture extension workers; CDOs, Community development officers.

 We conducted the open-ended interviews between February and June 2018 in Moroto and Namutumba. The interview guides are provided in Supplementary file 2. Consent was requested from all respondents to record the interviews. Interviews with frontline workers focused on four broad areas, synthesized from both nutrition and SLB literature (Table 1): (1) understanding of (mal)nutrition; (2) job descriptions, including nutrition actions; (3) individual experiences of providing nutrition services; and (4) nutrition-associated work demands and resources provided. Interviews were conducted at the workplace which permitted observation of the activities, work environment and interactions of frontline workers. Interviews with sub-county and district management and NGO actors prioritized the demands and resources issued and the frontline workers’ practices. This method enabled exploration of the workers’ experiences in their operational context and confirmation of narrations that were challenging to understand. Exit meetings were conducted with the district nutrition officers and NGO actors to verify the preliminary insights about the nutrition actions, work conditions and established routines. District nutrition officers coordinate planning and monitor implementation of nutrition actions across departments. To ascertain the demands and resources, interview data was complemented with insights from the local government policy reports. The first author previously supported policy development and implementation processes in Uganda. She leveraged the good professional network to access the study respondents, relevant documents and to observe service delivery. She was also able to engage in critical discussions and continuous peer review debates to question the similarities and differences in responses provided about the conditions and routines shaping the nutrition services.

 We transcribed and translated all the audio interviews into English to organize and analyze them using *Atlas.ti.* The first author repeatedly read the transcripts and coded the data based on the three thematic sensitizing ideas − nutrition actions, conditions, practices – described in operationalising street level bureaucrac. First, we coded the nutrition actions, using the categorization of specific and sensitive actions to identify the perceived services provided. Second, we coded the work conditions specifying action prescriptions and resources at individual, organization, and societal level. Third, we coded for practices if the data reflected the description of the patterns of practice explained under the SLB theory operationalised earlier. The coding categories were refined by comparing the extracted data to identify the recurring subthemes in each thematic area. For example, ‘pre-existing relationships’ and ‘non-involvement’ are additional codes that emerged inductively. The specific subthemes were used in progressive analysis. Practices were identified abductively. Authors continuously interpreted the empirical descriptions of routine actions of the frontline workers, and compared and embedded them in the language theorized in relevant implementation literature.^42^ For example, down prioritisation is a terminology used to indicate that services maybe offered but there is less focus on administrative tasks. The letters and numbers between parentheses refer to the district and respondents underpinning the empirical observation. Examples of illustrative quotes depicting each practice are included in Supplementary file 3.

## Results

###  Nutrition Services in Frontline Workers’ Everyday Work

 Most workers perceived nutrition services as the activities following from explicit nutrition projects of donors or government. Although their daily activities often comprised actions that according to literature^24-36^ are either nutrition-specific or nutrition-sensitive (Table 3), the workers did not always perceive them as nutrition actions. The nutrition services varied mainly among frontline workers in different departments. Further, the scope of these services differed depending on the districts and the departments’ historical involvement and present efforts to address malnutrition. Moroto proved to have a larger diversity of services compared to Namutumba. In both districts, health workers provided most nutrition services; this can be attributed to the fact that nutrition initiatives were traditionally delivered through the health system. Various AEWs and CDOs expressed uncertainty about their nutrition work and often considered their departments’ activities to be ambiguous. This is because most of their everyday tasks did not have explicit nutrition objectives.

**Table 3:** Nutrition Specific and Sensitive Services as Perceived by Frontline Workers

	**Moroto**			**Namutumba**		
	**HW**	**AEW**	**CDO**	**HW**	**AEW**	**CDO**
**Nutrition services **						
Anthropometric assessments	●			●		
Health and nutrition education	●			●		
Micronutrient supplementation	●			●		
Supplementary feeding programs	●					
Prescribing ready-to-use therapeutic foods	●					
Nutrition-related administrative actions (reporting)	●			●		
Promoting micronutrient-rich foods		●			●	
Distributing livestock		●				
Distributing planting material		●			●	
Community awareness of nutrition services			●			●
Distributing dry food rations			●			
Distributing planting material			●			
**Other nutrition services (but not perceived as such) **						
Iron folate supplementation (women)	●			●		
Anthelminthic control	●			●		
Distributing labor-saving technologies		●				
Demonstrating food-security production systems		●			●	
Distributing fruit trees		●			●	
Educating on post-harvest handling practices		●				
Public works for food purchase		●				
Household income support			●			
Women empowerment grants			●			●
Grants for the elderly			●			●

Abbreviations: HW, Health worker;AEW, agriculture extension worker; CDO, community development officer. ● The dot indicates the category of frontline workers offering the nutrition service.

###  Work Conditions Shaping Nutrition Service Delivery

####  Society-Related Conditions 

 The main societal-related conditions influencing nutrition practices were the donor requirements and the demands of citizens. Respondents emphasized the pivotal role of donor projects, especially funded by the United Nations Children’s Fund (UNICEF), the World Food Programme (WFP), and USAID, in shaping nutrition services in Uganda. These projects use different ideational and material resources to prescribe actions and support their implementation. This influence is asserted through three main mechanisms. First, donors provide technical support to district leadership to transpose national policies (eg, Uganda Nutrition Action Plan) and international agendas (eg, Sustainable Development Goals) into local programs and activities for implementation (N2, M34^[1]^). Second, donors issue directives stipulating procedures for implementing projects to standardize services, including targeted citizens, interventions and approaches used. Third, they constantly monitor specific performance indicators to catalyze responsiveness and ensure compliance by frontline workers. Donors projects employ different tactics to achieve compliance, including performance-based financing, supervision by contracted NGOs, and involving high level district management (M28, 34, 39, N9).

 A result of the donors’ central role in nutrition governance is that there were similarities in the nutrition services provided in both districts. Health workers, for example, explained that donors facilitate capacity building, print education materials, procure nutrition supplies (eg, therapeutic and supplementary foods) and equipment to ensure services availability (M28, N9). Actors, such as UNICEF, facilitate regular knowledge-sharing activities to foster collective learning and develop partnerships to realize mutual reinforcing nutrition objectives (M39). Given the high dependency on donor investments, nutrition service may be discontinued when this support stops, as observed in Namutumba.

 Other societal-related influences resulted from citizens’ increasing demands for and expectations about nutrition services. Historically, both government and donors distributed nutrition supplies and incentives (eg, farming inputs, food assistance) to manage malnutrition and encourage agriculture production, respectively. However, the free nutrition supplies are perceived to have encouraged dependency among citizens and increased demands for these ‘tangible’ services (N5, 17, 22, M11, 19).

####  Organization-Related Conditions

 In addition to donor conditionalities, frontline workers are increasingly issued action prescriptions and resources focused on nutrition from their ministries and associated departments especially health. Health workers identified two key action prescriptions from their superiors: (1) standardized guidelines for the management of acute malnutrition and (2) nutrition indicators integrated in the health management information system (HMIS). To realize these integrative demands, respondents stated that the ministry regularly collaborates with donor projects (NGOs) to build their capacities, conduct mentorships, and monitor nutrition services in health centers. Furthermore, pre-existing funded programs like the immunization program, provide avenues for providing nutrition actions (N20, 32). These prescribed actions explained the homogeneity of services provided across the health centers in both districts. However, health workers mentioned various ministry linked pitfalls that frustrate nutrition services, including the lack of explicit nutrition-focused budgets and the continued revisions made to the HMIS nutrition indicators without follow-up training (N32, M38, 40), constant human resources and skills transfers (N1, 21, 26) and inadequate feedback from superiors (N1, 20). Both AEWs and CDOs explained that their respective ministries lacked clear nutrition-linked performance indicators and guidelines.

 Leadership’s efforts to promote intra- and inter-departmental interactions proved an important resource enabling nutrition integration. The CDOs and AEWs in Moroto clarified that some sub-county leaders encouraged collaboration among departments and with NGOs hence presenting opportunities to network, learn about nutrition activities, and implement workplans (M13, 40). Health workers stated that the nutrition focal person facilitated quarterly learning meetings, funded by donors initiatives, where collective decisions were taken about the services offered (M2, 7, 16, 25). However, not all such interactions supported integration. In Namutumba, some CDOs and AEWs expressed discontent about the unclear allocation of duties in the multisector projects and the conflicting organization structures which frustrated the nutrition ambitions (N11, 15, 18).

####  Individual-Related Conditions

 Most demands experienced by frontline workers stemmed from the societal and organizational levels. Health workers repeatedly described the high workload and competing job tasks as determining prioritization. This undermined nutrition services in favor of ‘activities that have budget allocation and are incentivized’ (N15). In terms of resources, apart from existing expertise in managing undernutrition, nutrition services provision was influenced by expectation of auxiliary benefits, especially financial incentives. Nutrition was often considered an add-on activity, incentivized through project work (M34, N23). Hence, ‘the wide spread attitude that nutrition is a business is affecting integration’ (M33). Others indicated that their professional values, religious beliefs, and collaborations with colleagues (M1, 11, 25, N1, 20) were important drivers for providing nutrition interventions. That said, almost all AEWs and CDOs claimed not to have attended formal nutrition-focused training and thus typically derived any knowledge from work experiences (N8, M8) and learning from contemporaries in health and NGOs (M13).

###  Frontline Workers’ Practices Enacting and Inhibiting Nutrition Integration 

 To deal with the demands and resources discussed in the section above, frontline workers adopted various street-level practices that ultimately shaped service delivery. These practices reflect how nutrition services are generally organized and integrated into the frontline workers’ everyday tasks. Each group of frontline workers employed at least one practice (Table 4). The practices can be clustered along three categories: practices structuring access to nutrition services; practices controlling the supply of services; and practices modifying demand for services (Table 4).

**Table 4:** Practices Shaping Nutrition Service Delivery in Moroto and Namutumba Districts

**Practices **	**Moroto**			**Namutumba**		
	**HW**	**AEW**	**CDO**	**HW**	**AEW**	**CDO**
Structuring access to nutrition services						
Ritualizing performance of nutrition tasks	●			●		
Bundling nutrition actions with established services	●	●	●	●		
Scheduling nutrition services on a specific day	●					
Piggybacking onto other actors’ nutrition services		●	●		●	●
Controlling the supply of nutrition services						
Creaming off citizens	●		●	●		
Down prioritization of some nutrition services						
* fixating on a few nutrition actions*	●			●		
* non-involvement*					●	●
Modifying demand for nutrition services						
Shifting blame to other entities					●	●
Following the bureaucratic ‘jobs worth’				●		

Abbreviations: HW, Health worker;AEW, agriculture extension worker; CDO, community development officer. ● The dot indicates the frontline workers adopting the practice.

###  Practices Structuring Access to Nutrition Services

####  Ritualizing Nutrition Tasks Performance 

 The practice of ritualizing performance connotes following specific procedures and set routines, uniform decision making, and compliance with performance expectations in delivering nutrition actions.^28,43^ All health workers mentioned that they adhere to the prescribed procedures for identifying and managing acute malnutrition. These included ‘screening and categorizing children for malnutrition based set anthropometric cut-off points; enrolment into outpatient care (OTC) to receive therapeutic food, or hospital referral for inpatient care’ (M16). This practice resulted from following the ministry of health guidelines which articulate instructions about management of acute malnutrition. The practice is reinforced by NGOs through continuous capacity building and monitoring (N5, 20, M11, 22, 28). Ritualization of nutrition tasks is described as having standardized (and restricted) decision making and contributed to normalizing such services across health centers.

####  Bundling Nutrition Actions With Established Services

 Bundling involves aggregating nutrition actions with established services to leverage their resources and enhance simultaneous realization of complementary objectives.^44,45^ Although predominantly in Moroto, most health workers routinely bundled nutrition services with other daily tasks. This practice was usually spontaneous among AEWs and CDOs in Moroto. The main reasons for bundling by health workers include; (*i*) HMIS nutrition indicators being linked with pre-existing funded and regularly monitored programs (N34); (*ii*) development actors financing the implementation of particular combined services, ie, nutrition and HIV (M22); and (*iii*) donors, such as WFP, directives to ‘use the food rations to incentivize utilization of health service by pregnant women and lactating mothers’ (M38). Last, bundling by AEWs and CDOs arose out of the need to comply with instructions from the ministries (M8) and also compelled by their professional value to collaborate in delivering services.

####  Scheduling Nutrition Services on Specified Days 

 Scheduling refers to assigning a specific day on which citizens receive services for treating acute malnutrition.^46^ This practice was mainly mentioned by health workers in Moroto. Here, OTC services for malnutrition are offered on Thursdays (OTC or nutrition clinic day) (M2, 28). The OTC day incorporates screening citizens using stipulated criteria, dispensing therapeutic foods, and providing required health services. This way of organizing services helped to address the challenges of misappropriation of nutrition supplies, focus health workers’ prioritization of nutrition services and address pressure to achieve the donor performance benchmarks. Health workers explained that scheduling was collectively agreed to prevent citizens from ‘double-dealing and misusing therapeutic foods’ (M7, 16, 25). The practice became formalized through directives from UNICEF which restructured OTC services. Despite the high workload experienced on OTC day, scheduling is perceived to be beneficial in regularizing nutrition in health services, increasing malnourished case identification, and freeing-up heath workers’ time to attend to other activities (M2, 11, 18).

####  Piggybacking Onto Services Offered by Other Domains 

 Piggybacking refers to frontline workers depending on services already established by other actors to realize their nutrition objectives.^47^ It involves strategic collaborations, either between frontline workers in different sectors or with NGOs to realize mutual benefits,^48^ such as implementing workplans and securing the legitimacy of projects. The practice was demonstrated by AEWs and CDOs in both Moroto and Namutumba. Whereas piggybacking is a means of coping with disparities in – and often lack of – nutrition budgets, this practice was promoted by sub-county leaders to strengthen synergies across departments and with NGOs (M13); and by donors requiring NGOs to partner with government during project implementation (N22). Piggybacking is perceived to have improved the legitimacy of NGO activities (N30), enhanced the frontline workers’ understanding of nutrition (M13, 40, N14), and has financial benefits (M13, 8).

###  Practices Controlling the Supply of Nutrition Services 

####  Creaming off Citizens

 Creaming off means the prioritization of citizens to benefit from specified nutrition services, thereby restricting access to nonconforming ones.^10^ Although the practice was mentioned by most health workers, it was predominantly exhibited in Moroto. In addition to aligning with the criteria specified by the ministry for screening citizens with acute malnutrition, another reason for organizing services in this way was that most nutrition trainings emphasize particular citizen categories (N28, M25). Further, the frontline workers are strictly monitored by NGOs to ensure compliance with donor instructions (M38, 12, 16). Although nutrition assessments are conducted across all health facilities, there is priority focus on pregnant and lactating women, children and HIV or tuberculosis clients. For CDOs, creaming off is sporadic arising from one-off instructions, from the Office of the Prime Minister, which prioritize vulnerable community members to receive food rations.

####  Down Prioritization of Some Nutrition Actions 

 Down prioritization signifies the tactics employed to resist instructions issued to structure service delivery.^49^ This practice manifested in two distinct coping strategies: fixating on a few nutrition action and non-involvement. First,* fixating on a few nutrition actions *refers to frontline workers focusing on particular actions while ignoring others. This was common to health workers in both districts. The practice was expressed through varied behaviors, including restricting nutrition assessment to regularly monitored indicators (stunting, wasting and underweight) as compared to body mass index and laboratory based analyses. Other behavior was conducting group education sessions or encouraging citizens to read displayed information compared to individual counselling; and non-completion of administrative tasks. Incomplete nutrition data frustrates accounting for nutrition investments (M36, 38, N20, 34); and partly influenced donors to use performance-based financing to enforce compliance. Health workers explained that down prioritizing is inevitable because of the low staffing numbers (M19, N15). To manage the workload, they multitask or shift tasks to colleagues to offer – and often prioritize some – nutrition actions which compromises service completeness. Down prioritization was further enabled by the continual updates of HMIS nutrition indicators without follow-up training (M34, N8), perceived duplication of tasks done by NGOs such as record keeping (M12), and lack of financial nutrition incentives (N26).

 Second, *non-involvement *signifies withdrawal from participation in nutrition services because frontline workers perceive that their professional expertise and contribution are undermined and meaningless.^50^ The CDOs and AEWs were reluctant to engage in the nutrition activities implemented under the government-managed project in Namutumba. Majority indicated that this was a strategy to cope with the ambiguities in task allocation in this project and being side-lined in implementing services under their jurisdictions (N6, 19, 22). Although the practice is known to district management, they explained that its existence resulted from grievances over who controls budget expenditures and lack of performance incentives (N37). Conversely, through networking with colleagues in education, some AEWs supported the project activities whenever invited.

###  Practices Modifying the Demand for Nutrition Services 

####  Shifting Blame to Other Entities

 The practice of shifting blame indicates that the frontline workers blame their inability to provide nutrition services on other actors.^51^ Some AEWs, especially in Namutumba, felt incapacitated to provide nutrition services because delays in delivering farming inputs and the frequent mismatch between the citizens’ demands and supplied materials. The discrepancies were blamed on the perceived ‘inadequately designed bureaucratic procurement structure’ (N6) which undermines the AEWs’ professional expertise. The AEWs often reminisced about the past when they planned, procured, and distributed inputs based on farmers’ requirements. In addition, AEWs mentioned feeling demoralized by the citizens’ continued dependency on government for farming inputs compared to becoming self-reliant (N28, 33). An AEW explained that ‘many citizens are used to being given all the farming inputs and will only attend awareness-creation sessions if they know planting materials are to be distributed’ (N17).

####  Following the Bureaucratic “Jobs Worth”

 The ‘jobs worth’ practice refers to frontline workers rigidly following set rules to avoid confrontations with and blame from clients when their decisions result in negative effects.^51^ Health workers normally refer acutely malnourished citizens according to the ministry guidelines. However, this referral for upward management is a prioritised action to limit pressure from citizens who demand for ‘tangible services’ (eg, therapeutic foods) and to minimize perceptions of negligent treatment. This practice was rigidly employed to cope with the lack of nutrition supplies (eg, therapeutic foods, micronutrient supplements). Health workers in Namutumba were frustrated by the perceived ‘reduced authenticity of nutrition services’ caused to the lack of supplies; which was attributed to the high turnover in donor projects and insufficient clarity on how nutrition support in the district is organized (N4, 9, 20). Thus, by pushing citizens presenting with any severe malnutrition to the general hospital, they transfer the ‘responsibility of ensuing repercussions back to the citizens and upward to the health system’ (N10). Moreover, the number of citizens seeking and accessing these nutrition services remains unascertainable.

## Discussion

 We started with the observation that integrating nutrition actions into service delivery in different policy sectors is a continuous concern.^5^ We proposed that adopting the SLB approach offers novel bottom-up viewpoints about what practices frontline workers in different departments adopt to enact or impede nutrition integration in service delivery, and the contextual conditions galvanizing them. This study indicates that nutrition integration into service delivery is predominantly perceived as the task for health workers compared to AEWs and CDOs. Frontline workers’ perception of what constitutes nutrition work in their everyday activities was not necessarily aligned with the conceptualization of nutrition-specific and nutrition-sensitive actions found in the nutrition literature.^24-36^ Our analysis uncovered nine practices adopted by frontline workers that ultimately affect the ways that nutrition services are delivered to citizens. Nutrition integration into delivery systems is commonly accomplished through ritualizing task performance; bundling with established services; scheduling services on specified days; and piggybacking onto services offered by other domains. Disintegration results from non-involvement and shifting blame to other entities. Three of the identified practices potentially have both integrative and disintegrative effects: creaming off citizens; down prioritization by fixating on a few nutrition actions; and following the bureaucratic ‘jobs worth.’

 This study illustrates that integrative/disintegrative practices co-exist and potentially bolster and/or inhibit one another’s effects. This implies that frontline workers’ practices are shaped not only by contextual demands and resources, but also by the ongoing interactions among them.^52^ For example, ritualizing task performance underlies the bundling of nutrition actions with established services; while shifting blame to others reinforces non-involvement. Further, there are similarities and differences in the practices of frontline workers in comparable departments, but located in different contexts. All health workers use bundling to incorporate nutrition services. However, scheduling of nutrition actions and creaming off citizens were demonstrated only in Moroto. Equally, piggybacking was common among all AEWs and CDOs, but non-involvement only happened in Namutumba. This can possibly be explained by the disparities in resources, ambiguities in roles for providing nutrition services, and variation in nutrition demands between the districts. This finding echoes observations that advances in integrating nutrition activities in health and agriculture services are predisposed to contextual differences.^9,11^

 The individual, organization, and society demands and resources influencing frontline workers are cited in other nutrition literature for example^11,53^, although these conditions are seldom patently linked to integrative/disintegrative practices. This analysis provides three main insights about the circumstances influencing nutrition services. First, integrative practices are mainly driven by donors (eg, UNICEF, WFP, USAID). In Moroto, donors concurrently used varied ideational and material resources, such as directives of services offered and nutrition supplies, respectively. These resources structured the nutrition services in ways that aligned with the donors’ ideologies and interests. Further integrative practices were ensured through financing capacity development, and monitoring compliance using different ‘carrot and stick’ tactics like performance-based financing. These strategies resulted in all health workers specializing in and standardizing services for managing undernutrition. This finding signals that NGOs leverage different resources − including problem framing, rulemaking, brokering alliances, and financial and political resources – as they negotiate and maneuver to institutionalize services to attain project objectives.^54^ Donor conditionalities may continue to structure services beyond the funding period, as observed in Namutumba; nonetheless, the sustainability of integrative practices remains unascertainable.^55^

 Second, demands and resources institutionalized in the government systems are necessary to sustain nutrition service delivery beyond the short-term donor projects. The study offers insights to support the calls to specify intervention pathways and performance measures per sector, strengthen capacities, and foster effective leadership to facilitate nutrition policy implementation.^56^ Conversely, this analysis also reveals that unclear allocation of responsibilities, narrowly defined objectives and performance indicators restrict the scope of nutrition services, thereby facilitating down prioritization practices.^10^ For example, no district offered services to manage overnutrition. Further, frontline workers operating in resource-constrained environments (ie, limited government funding, inadequate technical capacities, ambiguous nutrition-sensitive actions and reporting systems) will maneuver to redirect responsibilities to other actors,^28,51^ hence shifting blame to others.

 Third, the frontline workers’ professional relationships and performance rewards are equally as important as their expertise in persuading them to provide nutrition services. Besides the workload and labor‐intensive tasks associated with integrative demands,^31^ disintegrative practices, like down prioritization, were associated with the negative impact of financial incentives. Most frontline workers perceived ‘nutrition as a business’ that rewards good performance. Nonetheless, financial incentives are not a sufficient condition to maintain their commitment, partly due to variation in donors’ facilitation approaches.^10^ Our study shows that professional collaborations presented beneficial opportunities for AEWs and CDOs to learn informally about nutrition services, and this supported integrative practices such as piggy-backing and scheduling activities on fixed days.

 The intricacies involved in integrating nutrition into service delivery raises some questions for further research. First, the identified practices are for frontline workers in government bureaucracies; however, nutrition services are also provided by employees of non-governmental and private sector organizations. Understanding their nutrition integrative/disintegrative practices is essential to comprehend the internal dynamics (ie, demands, resources, strategies, and practices) of how nutrition integration is realized across all actors involved; and inform efforts to translate policy statements into real action and impact on the ground.^52,54^

 Second, the practices identified are not static; thus, future research could explore the changes in frontline workers’ practices over time and space. This synthesis provides preliminary insights into integrative/disintegrative practices beyond the health sector, but see Fanzo et al.^9^ Additional research employing a similar SLB perspective is necessary to highlight the extent to which and how contextual changes − in terms of different time periods, technical and geographic boundaries – shape frontline workers’ practices and the resultant effect on nutrition service delivery.

 Third, using the SLB perspective reveals new aspects of micro-contextual interactions and how they shape nutrition policies as practiced on the ground. However, this analysis is restricted to Uganda. It would be worthwhile to use this public policy lens to perform comparative analyses across other low-income countries. This could provide further insights into what needs to be done to generate the desired policy outcomes that ensure that nutrition services are available to citizens that need them.

 Lastly, the analysis suggests that nutrition integration/disintegration on the ground is a dynamic and nonlinear process^57^; however, identified practices may become ingrained norms over time. These practices have considerable, possibly detrimental, implications for citizens. For instance, there are differences in scope of services and what citizens consider to be useful nutrition actions. Research should examine the effects of frontline workers’ practices from the citizens’ perspective.

 From this study’s findings, three governance implications for improved nutrition integration during service delivery can be made. First, there is a need to harness the integrative-fostering capacity of all frontline workers, beyond the health sector. This analysis identifies various strategies that generated integrative practices among frontline workers, and these are potentially transferable for application in different contexts.^58^ Strategies such as identifying relevant interventions, collective learning and consensus building, shadowing during service provision, developing integrative leadership, and collaborations among frontline workers − when appropriately understood provide insights into how to strengthen integration on the ground in different policy sectors.

 Second, similar to national level policy processes,^16,36,55^ nutrition integration in service delivery is propelled mainly by the presence and push of donors. However, these actors have varied interests and thus prescribe unilateral directives to frontline workers that may increase discrepancies in integrative demands during service delivery. This does not imply that the strategies should be rejected, but rather that it is necessary to continuously develop capacity and invest in state-driven service delivery systems to facilitate and sustain nutrition policy implementation. Government institutions are usually resource constrained which limits integration in the short term, however these domestic systems are important to sustain nutrition integration in the long term.

 Third, our analysis shows that there are variations in what AEWs and CDOs label as nutrition services. Some services perceived as usual government business (Table 3) are potential opportunities for integrating nutrition services. The fact that there are ambiguities in articulating nutrition-sensitive actions and in the roles of AEWs and CDOs demonstrates the necessity of generating consensus around key strategic actions in these policy areas, with explicitly defined pathways linking them to nutrition outcomes.^56^ Elaboration of what integrated government action on the ground actually means is essential for the propagation and sustainability of nutrition activities across workers in different ministries.

## Conclusion

 This study started with the question of what practices frontline workers in different departments adopt to enact or impede nutrition integration in service delivery and the contextual conditions galvanizing them. The SLB approach, which is underutilized in integrated policy action studies, provided essential insights about the micro-dynamics defining integrated government action for nutrition. Examining the diverse demands and resources arising from diverse individual, organizational, and societal contexts demonstrates that frontline workers adopt varied practices; which possibly explain the inconsistencies between policy goals and actual outcomes.^5^ Donor initiatives are essential in facilitating nutrition integration into service delivery. However, the study emphasizes that negotiation and collective understanding of demands, legitimizing responsibilities, matching performance accountability with equivalent support (resources), developing integrative capacity at subnational level, and fostering professional collaboration are vital to sustain these efforts. These are long-term endeavors − not easily sustained through short-term funded projects − that necessitate going beyond tracking the adoption of integrated strategies and checking off performance indicators. As governments and international actors continue to make commitments to effectively improve nutrition outcomes and to develop sustainable food systems, understanding the integrative practices of frontline workers provides an essential starting point to identify effective policy solutions.

## Acknowledgements

 The authors acknowledge funding for this research from NUFFIC, the Dutch Organization for Internationalization in Education.

## Ethical issues

 The approval to conduct this study was obtained from the Uganda National Council of Science and Technology through permit number SS83EB and the Mild May Uganda Research Ethics Committee under # REC REF 0160-2017.

## Competing interests

 Authors declare that they have no competing interests.

## Authors’ contributions

 BSN: conception and design, acquisition of data, analysis and interpretation of data, drafting the manuscript, statistical analysis, administrative, technical and material support. JJLC: conception and design, analysis and interpretation, critical review of manuscript, supervision. KJAMT: critical review of manuscript, obtained funding, supervision. EFT: conception and design, analysis and interpetation of data, critical review of manuscript, supervision. JH: conception and design, analysis and interpretation of data, critical review, administrative, technical and material support.

## Funding

 This works was supported by NUFFIC, the Dutch Organization for Internationalization in Education [Grant number: NFP-PhD 16/0019].

## Endnotes


^[1]^ The parenthesized numbers refer to respondents’ empirical observations in Moroto (M...) or Namutumba (N...).

## Supplementary files


Supplementary file 1. Sampling Approach.

Supplementary file 2. Interview Guide for Frontline Workers.

Supplementary file 3. Illustrative Quotes of Practices Shaping Nutrition Service Delivery.
