# Supplementary material for: Integrating Nutrition Actions in Service Delivery: The Practices of Frontline Workers in Uganda
Source: Int J Health Policy Manag. 2022 Apr 24;11(12):2895–906. doi: 10.34172/ijhpm.2022.5898 (PMC10105165; doi:10.34172/ijhpm.2022.5898)
Supplement: Supplementary file 1 — Sampling Approach. [file ijhpm-11-2895-s001.pdf]

Article title: Integrating Nutrition Actions in Service Delivery: The Practices of Frontline Workers in Uganda

Journal name: International Journal of Health Policy and Management (IJHPM)

Authors' information: Brenda Shenute Namugumya[1]*, Jeroen J.L. Candel[1], Elise F. Talsma[2], Catrien J.A.M. Termeer[1], Jody Harris[3]

[1]Public Administration and Policy Group, Wageningen University & Research, Wageningen, The Netherlands.

[2]Division of Human Nutrition and Health, Wageningen University & Research, Wageningen, The Netherlands.

[3]Institute of Development Studies, University of Sussex, Brighton, UK.

(corresponding author: brenda.namugumya@wur.nl)

Supplementary file 1. Sampling Approach

At the time of data collection, both Moroto and Namutumba had six sub-counties where nutrition services were offered. Data was collected in all the sub-counties.

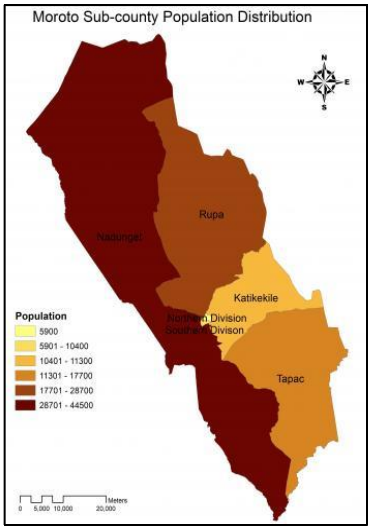

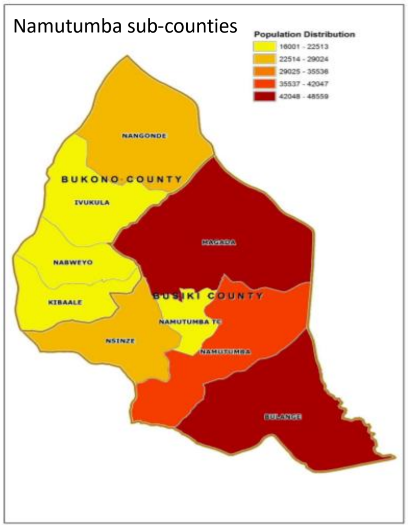

Source: Uganda Bureau of Statistics 2014

Moroto district

Sub counties: 6 - Rupa, Katikekile, Nadunget, Northern Division, Southern Division, Tapac

- Total number of health facilities: 19

  # Regional referral hospital (RRH): 1

Health Centre III: 6

Health Centre II: 12

Based on district health service records, nutrition services are provided at the RRH, all HC IIIs and a limited number of HC IIs (n=5). Interviews conducted for all frontline workers engaged in delivering nutrition services in the health facilities (n=12).

- Agriculture extension officers (n=6) and community development officer (n=6). All were interviewed, except the two that were out of office.

Namutumba district

Sub counties: 6 - Ivukula, Kibaale, Magada, Nsinze, Namutumba, Bulange

- Total number of health facilities: 33

  # Regional referral hospital (RRH): none

  # Health Centre III: 6

  # Health Centre II: 26

  Nutrition services were offered at all HC IIIs and 8 HC IIs (majority of the HC II were not in full operation). The health centres were selected in consultation with the district health officer (n=14).

- Agriculture extension officers (n=6) and community development officers (n=5). Some respondents were out of office, and two declined to participate in the interviews.

District and subcounty management

The subcounty leadership (subcounty chief) for all sub counties were interviewed, except one that declined in Namutumba. At district level, the respective department heads for agriculture, health and agriculture and the Chief Administrative Officers were interviewed.

Nutrition projects

The nutrition staff of all the projects offering nutrition services in the districts were interviewed.
