# Supplementary material for: Integrating Nutrition Actions in Service Delivery: The Practices of Frontline Workers in Uganda
Source: Int J Health Policy Manag. 2022 Apr 24;11(12):2895–906. doi: 10.34172/ijhpm.2022.5898 (PMC10105165; doi:10.34172/ijhpm.2022.5898)
Supplement: Supplementary file 2 — Interview Guide for Frontline Workers. [file ijhpm-11-2895-s002.pdf]

Article title: Integrating Nutrition Actions in Service Delivery: The Practices of Frontline Workers in Uganda

Journal name: International Journal of Health Policy and Management (IJHPM)

Authors' information: Brenda Shenute Namugumya[1]*, Jeroen J.L. Candel[1], Elise F. Talsma[2], Catrien J.A.M. Termeer[1], Jody Harris[3]

[1]Public Administration and Policy Group, Wageningen University & Research, Wageningen, The Netherlands.

[2]Division of Human Nutrition and Health, Wageningen University & Research, Wageningen, The Netherlands.

[3]Institute of Development Studies, University of Sussex, Brighton, UK.

(corresponding author: brenda.namugumya@wur.nl)

Supplementary file 2. Interview Guide for Frontline Workers

<u>Background information of respondent</u>

What type of sector do you work for?
Health ☐; Agriculture ☐; Community development ☐; Other(specify)

What type of institution / organisation do you work for?
Government ☐; NGO ☐

What is your profession? ____

Your years of working experience in the sector/organisation.
1-5yrs ☐; 6-10yrs ☐; 11-15yrs ☐; 16-20yrs ☐; Over 20yrs ☐

Your years of experience in nutrition service delivery.
1-3yrs ☐; 4-6yrs ☐; Over 6yrs ☐

Female ☐ Male☐

<u>Job specific attributes</u>

1. How do frontline workers (e.g. health workers, extension workers, community development officers, teachers) execute nutrition related actions?
    a. What are the specific tasks (according to job description) performed by the frontline workers? *(probe: what do frontline workers see as their usual task)*
    b. What kind of nutrition activities are most important for frontline workers to perform? *(probe: nutrition challenges in the communities, understanding of services integration)*

c. How do frontline workers solve nutrition related problems in their day to day actions? (*probe: What nutrition services do you usually provide? Why?; how do they make decisions*)
d. What influences the actions of frontline workers? *(probe: who decides what frontline workers should do in nutrition; where did they learn the skills, what professional norms are specific in this line of work)*
e. What relationships or interactions exist among frontline workers? (*probe: interactions other frontline, levels of governments, NGOs, information networks*)

Situational factors

2. What is the role of socio-political and organisation (e.g. management) context on decisions and practices of frontline workers?
   a. What specific situations influence the decisions and practices of frontline workers? (*probe: role of politics, events in communities, role of NGOs, influence of administration systems*)
   b. What are the official goals for provision of nutrition services? *(probe: who sets these goals)*
   c. Which pressures do the different frontline workers experience from within and outside their organisation that influence nutrition services? *(probe: how do they deal with these pressures)*
   d. To what extent do expectations of peers influence nutrition decisions made by frontline workers? *(probe: which actors influence decision making, how)*
   e. Who support the nutrition services that you undertake?
   f. What are the incentives for provision of nutrition services? (probe: *What carrots and sticks accompany how frontline workers provide/not provide nutrition services?*)
   g. What management systems are influencing the decisions and practices of frontline workers? (*probe: systems in government and non-government, ask for guiding policy documents*)

Individual Characteristics

3. What is the role of individual characteristics on the decisions and practices of frontline workers?
   a. What capacity building initiatives have frontline workers participated in to enhance their knowledge in nutrition? (*probe: length of training, work experience, personal characteristics*)
   b. How has this exposure influenced the way you provide nutrition services?
   c. What professional norms do frontline workers have about delivery of nutrition services? (*where do these values come from, how does if influence their behaviour*)
   d. Which agencies/actors do you interact with in the provision of nutrition services? Which non-governmental organisations do are engaged in nutrition policy implementation in your community

Interview guide District/Organisations

1. What is your (organisation) understanding of the Uganda nutrition action plan (nutrition policy)? (*probe: What is the policy about? How has it been interpreted in the organisation? What does this policy mean to the daily practice of various frontline workers?)*
2. What is the role of socio-political and organisation (e.g. management) context on decisions and practices of frontline workers?
    a. What organisation conditions are influencing the nutrition practices of frontline workers? (*probe: conditions in government and non-government systems, health, agriculture,*)
    b. What are the incentives for provision of nutrition services? (probe: *What carrots and sticks accompany how frontline workers provide/not provide nutrition services?*)
    c. What specific situations have influenced the decisions and practices of frontline workers engaged in nutrition? (e.g. *role of NGOs, influence of administration systems*)
3. How is your organisation influencing nutrition service delivery in sub-counties?
    a. What is your/organisation understanding of integrated nutrition service delivery?
    b. How has this understanding shaped nutrition services provided by frontline workers (health, agriculture, community development)?
4. What changes have occurred over the past 10 years (2009-2019) that have influenced nutrition programing/interventions in this district? *(on a timeline probe: role of politics, events in the communities, management related, role of NGOs)*
    a) How have these changes shaped the delivery of nutrition services?
    b) Which pressures do the different frontline workers experience from within and outside their organisation that influences nutrition services? *(probe: organisation performance monitoring, how do they deal with these pressures)*
    c) What management systems are influencing the decisions and practices of frontline workers? (*probe: systems in government and non-government, ask for guiding policy documents*)
5. How has commitment for nutrition been generated in the sub-counties?
6. What challenges are faced in ensuring the integration of nutrition in services provided by different frontlines in the district?
