# Supplementary material for: Integrating Nutrition Actions in Service Delivery: The Practices of Frontline Workers in Uganda
Source: Int J Health Policy Manag. 2022 Apr 24;11(12):2895–906. doi: 10.34172/ijhpm.2022.5898 (PMC10105165; doi:10.34172/ijhpm.2022.5898)
Supplement: Supplementary file 3 — Illustrative Quotes of Practices Shaping Nutrition Service Delivery. [file ijhpm-11-2895-s003.pdf]

**Article title:** Integrating Nutrition Actions in Service Delivery: The Practices of Frontline Workers in Uganda

**Journal name:** International Journal of Health Policy and Management (IJHPM)

**Authors' information**: Brenda Shenute Namugumya[1]*, Jeroen J.L. Candel[1], Elise F. Talsma[2], Catrien J.A.M. Termeer[1], Jody Harris[3]

[1]Public Administration and Policy Group, Wageningen University & Research, Wageningen, The Netherlands.

[2]Division of Human Nutrition and Health, Wageningen University & Research, Wageningen, The Netherlands.

[3]Institute of Development Studies, University of Sussex, Brighton, UK.

(corresponding author: brenda.namugumya@wur.nl)

**Supplementary file 3.** Illustrative Quotes of Practices Shaping Nutrition Service Delivery

| Practice | Illustrative quotes |
|---|---|
| **Ritual performance of nutrition tasks** | 'We screen all OPD clients, the children with malnutrition - if they are in the red or yellow (MUAC) we admit them into OTC program and give them plumpynut (therapeutic food) for treatment. Depending on their condition, they are usually admitted for three months; ....we discharge and refer them to supplementary feeding and when they recover, we give them minerals and vitamins powder to take home'. M18<br>'We conduct assessment, classification, and then health and nutrition talk. Nutrition assessment is integrated in all the departments that show some influxes in clients..... Those who are malnourished, we refer them to Iganga hospital for treatment, whether in OTC or inpatient care'. N2 |
| **Bundling nutrition actions with established services** | 'We assess clients for nutrition parameters whenever they come because it is a requirement. We take the weight, height, mid upper arm circumference and give nutrition education sessions. This is the routine during the triage before getting progressive services. This is done in all our clinics like outpatient department (OPD), maternity, immunisation and HIV/TB. For inpatients, we do not really take it as important because clients pass through OPD and are usually not kept here for long'. N9<br>'Most people used not to come to health centres for the services..... That is why food distribution occurs at HC IIIs that implement the Maternal Child Health and Nutrition program (MCHN)sponsored by WFP. Food is an incentive for pregnant women and lactating mothers to come for antenatal care, maternity and immunisation services to prevent malnutrition. This has greatly increased coverage and utilisation of MCHN services'. M38<br>'We integrate nutrition during community dialogues........when we see children who are malnourished, we tell the parents how to make sure that child is helped. We tell them to go to hospital and there are very many partners that are helping us ensure that those malnourished children are helped like'. M14 |

| | |
|---|---|
| **Scheduling nutrition services on particular days** | 'Like for acute malnutrition management, they have clinic days which is usually Thursday, where they expect all severely malnourished children to come for care that day, just like you may have an HIV clinic day. So on that OTC/ nutrition day, you would really expect to have many people because that is their appointment date and they have to come to pick their treatment.'M22 |
| **Piggybacking onto nutrition services in other domains** | 'There is a lot of interaction because when we do agriculture work, there are cross cutting components, where we collaborate with health and CDO. It is actually encouraged by leadership. Our leadership encourages us to work in synergies. It is also about self-respect and following the code of conduct at work, It is about sharing the cake'. M8<br>'As a government [CDO] we do not have activities related to nutrition. Instead we work with partners [NGOs] and are involved in creating awareness. We go to them, but others come to us directly to participate in their activities, mostly like create awareness on nutrition, mostly. Nutrition is important to some of our friends here [partners] so we try to integrate because we are always in meetings about them'. M9 |
| **Creaming of clients** | 'We attach food to services mainly targeting pregnant women and lactating mothers to encourage then to come for antenatal visits, delivery in the health facility and to bring children for immunisations'. M40<br>'We usually screen children for malnutrition, and those who meet the criteria are enrolled into OTC to receive plumpynut (Therapeutic food). The ones who recover are referred to AFI for supplementary feeding.......'M25 |
| **Down prioritisation: fixating on a few nutrition actions** | 'We decided to plot graphs showing our work so that in case anyone needs the information they can look at the charts and know what they want. Sometimes we are busy and so when they come, they register and take information displayed on the walls. Also it makes reporting easy for us and with assessments, we get through the process quickly'.M4<br>'Our patients are assessed for nutrition parameters, their weight, height, MUAC, are routine for almost all children. We would love to do BMI for others but time is not enough.......When the client load is too high, there are somethings that we may not prioritise such as assessment and data entry. You may want to do all those things but at times we are so few. The high workload make is difficult so you either miss out or weigh with challenges and may not record...' N15 |
| **Down prioritisation: non-involvement** | 'There is a nutrition project in schools.... which gives some money to them to make sure that they grow some food especially vegetables.....Production is normally expected to train on how gardens are set up, supervise and monitor how everything is running and submit a report to the coordinator. But personally I haven't received the training, the project has its own trainers introduced to the schools.... So our role is not clear and yet we are expected to report'. N17 |
